# Synthetic Methane-Consuming Communities from a Natural Lake Sediment

**DOI:** 10.1128/mBio.01072-19

**Published:** 2019-07-23

**Authors:** Zheng Yu, Joseph Groom, Yue Zheng, Ludmila Chistoserdova, Jing Huang

**Affiliations:** a Department of Chemical Engineering, University of Washington, Seattle, Washington, USA; University of British Columbia

**Keywords:** methane oxidation, synthetic community, methanotrophs, species interactions

## Abstract

The factors and processes that influence the behavior and functionality of ecosystems inhabited by complex microbiomes are still far from being clearly understood. Synthetic microbial communities provide reduced-complexity models that allow an examination of ecological theories under defined and controlled conditions. In this study, we applied a multiphasic approach to study synthetic methane-oxidizing communities and species interactions as proxies to the natural communities. Our results confirm that, under selective pressures, natural-sediment communities of high complexity simplify rapidly, selecting for several major functional guilds, the major partners in methane oxidation being the *Methylococcaceae* methanotrophs and the *Methylophilaceae* methylotrophs, along with minor but persistent partners, members of *Burkholderiales* and *Flavobacteriales*. As a proof of concept, we established minimalist synthetic communities that were representative of the four functional guilds to demonstrate the dependency of the non-methane-utilizing species on the methanotrophs as the primary carbon-providing species. We observed that in communities consisting of multiple representatives of the key guilds, members of the same guild appeared to compete for resources. For example, when two methanotrophs of the same family were present, the two expressed similar key methanotrophy pathways and responded similarly to changing environmental conditions, suggesting that they perform a similar keystone function *in situ*. Similar observations were made for the *Methylophilaceae*. However, differences were noted in the expression of auxiliary and unique genes among strains of the same functional guild, reflecting differential adaptation and suggesting mechanisms for competition. At the same time, differences were also noted in the performances of partners with specific metabolic schemes. For example, a mutant of Methylotenera mobilis impaired in nitrate utilization behaved as a more efficient cooperator in methane consumption, suggesting that the loss of function may lead to changes in communal behavior. Overall, we demonstrate the robust nature of synthetic communities built of native lake sediment strains and their utility in addressing important ecological questions while using a simplified model.

## Abstract (precis)

The metabolism of methane is an important part of the global carbon cycle. While deciphering the community function and the potential role of the different functional guilds is very difficult when considering native complex communities, synthetic communities, built of species originating from a study site in question, present a simplified model and allow specific questions to be addressed as to carbon, nitrogen, and other nutrient transfer among species in a controlled system. This study applies an ecophysiological approach, as a proof of principle, to an already well-studied model system, contributing to a better understanding of microbial community function and microbial ecosystem processes.

## INTRODUCTION

Methane is one of the major contributors to climate change, with its atmospheric concentration steadily increasing over the past 300 years, mostly due to anthropogenic activities (1–3). Lake sediments are environments that are both major sources and major sinks of methane (4). Lake Washington sediment has served as a study site for studying methane-oxidizing bacteria for decades (5), especially since the onset of the use of molecular tools for environmental detection, including metagenomics (6–9). The major functional guild of microbes involved in methane consumption in these environments is the aerobic methane-oxidizing bacteria, whose activity constitutes a natural filter that mitigates the escape of methane into the atmosphere (10). However, the factors and processes that influence the behavior and functionality of a methane-oxidizing microbiome are hard to elucidate when using *in situ* experiments due the complexity of the natural communities, the unpredictable changes in community structure due to uncontrollable factors such as predation, the heterogeneity of organisms representing major functional guilds, and the interconnected nature of biogeochemical cycles. In this regard, a model synthetic system that links function to identity can help elucidate the relationships between microorganisms and ecosystem processes (11, 12).

Laboratory-cultivated synthetic cocultures represent a simplified approach to assessing interactions among different species, allowing for their controlled manipulation and for detailed analysis of the individual strains involved (13). Specifically, for methane utilization in nature, the traditional model of methanotrophs acting alone in oxidizing methane to CO_2_ and assimilating formaldehyde into biomass (14) may in fact be simplistic. Instead, it appears that communities rather than single species are involved, and carbon is transferred from the methanotrophs to nonmethanotrophs (15, 16). So far, strong evidence has been presented that methanotrophs of the family *Methylococcaceae* provide significant amounts of carbon to other community members, one of the most persistent partners being nonmethanotrophic methylotrophs of the family *Methylophilaceae* (17).

In this study, we expanded analyses to include additional functional guilds involved in methane-utilizing communities. We first revisit complex bacterial communities inhabiting natural Lake Washington sediment and compare them to communities simplified by selective pressure during incubation under methane, identifying *Burkholderiales* and *Flavobacteriales* as persistent members of methane-oxidizing communities. We then built synthetic communities by employing pure cultures of bacteria representing the following four major functional guilds: methanotrophs of the family *Methylococcaceae*, nonmethanotrophic methylotrophs of the family *Methylophilaceae*, and nonmethanotrophic heterotrophs of the families *Comamonadaceae* and *Flavobacteriaceae*, all isolated from Lake Washington sediment. The minimalist synthetic community models investigated included four to 14 species. To understand the function, partnership, and role of competition in these synthetic communities, flow cytometry, metagenomics, metatranscriptomics, and stable isotope DNA labeling were employed.

## RESULTS AND DISCUSSION

### Shifts in natural community composition identify additional functional guilds that respond to the methane stimulus.

**FIG 1:**
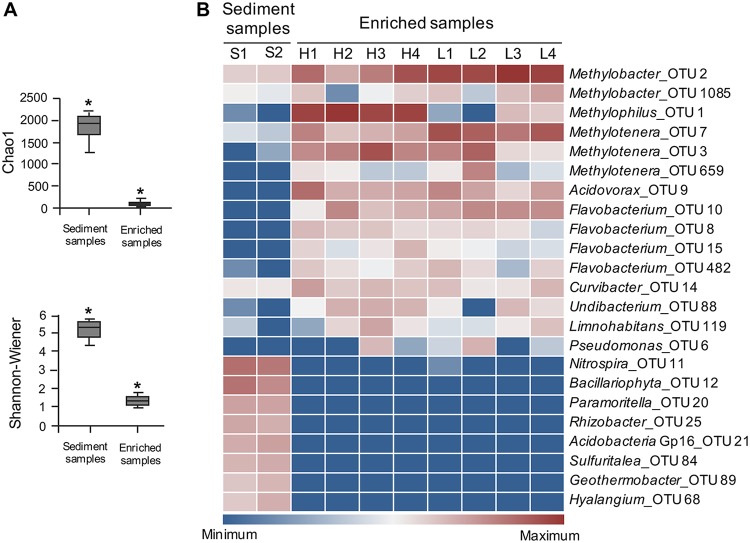
Composition of natural Lake Washington sediment community and communities enriched in response to methane stimulus. Data from reference 18, representing samples taken after 4-week enrichment, were reanalyzed. Only species represented by the dominant operational taxonomic units (OTUs; abundance, ≥1%) are displayed. (A) Diversity indices differ significantly between natural and enriched samples. Analysis of variance (ANOVA) was used in combination with Scheffe’s F multiple-comparison test to examine differences among the samples. (B) Heatmap reflecting abundances of major OTUs in natural and enriched communities. H, high dioxygen concentration; L, low dioxygen concentration; 1 to 4, replicate samples. For further details, see reference 18.

We revisited the previously generated data sets from natural Lake Washington sediment and laboratory microcosms manipulated under conditions of variable dioxygen availability (18). In addition to the previously noted dominant community members, the *Methylococcaceae* and the *Methylophilaceae* (18–20), we noted the minor but persistent presence of two additional functional guilds, bacteria of the orders *Burkholderiales* and *Flavobacteriales*, suggesting that carbon from methane may be transferred beyond *Methylophilaceae* methylotrophs to a broader community (Fig. 1).

### Isolating representatives of *Burkholderiales* and *Flavobacteriales* as prospective members of methane-oxidizing communities.

To test the propensity of *Burkholderiales* and *Flavobacteriales* to participate as members of methane-oxidizing communities, we carried out enrichment and isolation experiments as described in Materials and Methods. We were able to isolate multiple strains of *Burkholderiales* and *Flavobacteriales* from methane enrichment cultures by selecting them on R2A medium. None of these organisms were able to grow on either methane or methanol (not shown), suggesting that, likely, multicarbon substrates excreted by either *Methylococcaceae* or *Methylophilaceae* or both were supporting their growth. Several strains (see Table S1 in the supplemental material) were employed as parts of the synthetic communities in this study.

10.1128/mBio.01072-19.1 TABLE S1 Strains used in this study, with respective characteristics. Download Table S1, DOCX file, 0.1 MB. Copyright © 2019 Yu et al. 2019 Yu et al. This content is distributed under the terms of the Creative Commons Attribution 4.0 International license.

### Establishing multispecies minimalist synthetic communities from native isolates.

**FIG 2:**
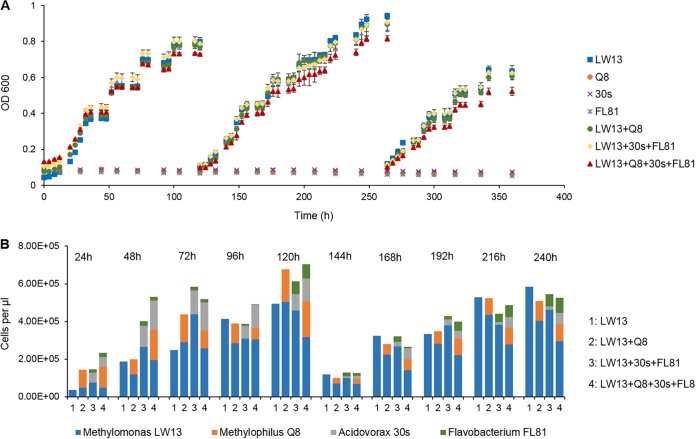
Growth and population abundances in synthetic communities. (A) Optical densities at a wavelength of 600 nm measured over time. At the 120- and 264-h points, cultures were diluted 10-fold in a fresh medium. Error bars indicate standard deviation (*n *=* *3). (B) Flow cytometric cell counts taken at 10 time points.

As a proof of concept, we established cocultures of organisms representing the four major functional guilds implicated in methane consumption, the bona fide methanotrophs of the *Methylococcaceae* family, methylotrophs of the *Methylophilaceae* family, and nonmethanotrophic heterotrophs of the *Burkholderiales* and *Flavobacteriales*, as a novel synthetic model community system, fed with methane as the sole carbon source (Fig. 2).

A prototypical minimalist community was built of four strains, Methylomonas sp. strain LW13, Methylophilus methylotrophus Q8, Acidovorax sp. strain 30s, and Flavobacterium sp. strain 81, and this mixture was cultivated in a mineral salts medium with methane as the sole carbon source. Single-strain cultures were used as controls. The optical densities and cell counts (measured daily) of each species in cocultures steadily increased. The pure culture of *Methylomonas* sp. LW13 showed the highest optical densities compared to other coculture treatments (Fig. 2A). The four-strain mixtures grew significantly slower than did the others, especially after two transfers with dilutions near the end of the experiment (Fig. 2A). Figure 2B shows individual strain cell counts as determined by flow cytometry, measured daily. Over the course of the experiment, the abundances of the four populations fluctuated over time, resulting in population ratios ranging from 20:80 to 50:50 (*Methylomonas* sp. LW13 to *M. methylotrophus* Q8 plus *Acidovorax* sp. 30 and *Flavobacterium* sp. 81) over the course of the experiment. As predicted, the nonmethanotroph partners (*M. methylotrophus* Q8, *Acidovorax* sp. 30s, and *Flavobacterium* sp. 81) did not grow on methane as the sole source of carbon (Fig. 2A). In support of the hypothesis of carbon transfer from *Methylomonas* spp. to other community members, the cell counts of *Methylomonas* sp. LW13 in the cocultures were significantly lower than those observed in the pure culture (Fig. 2B).

**FIG 3:**
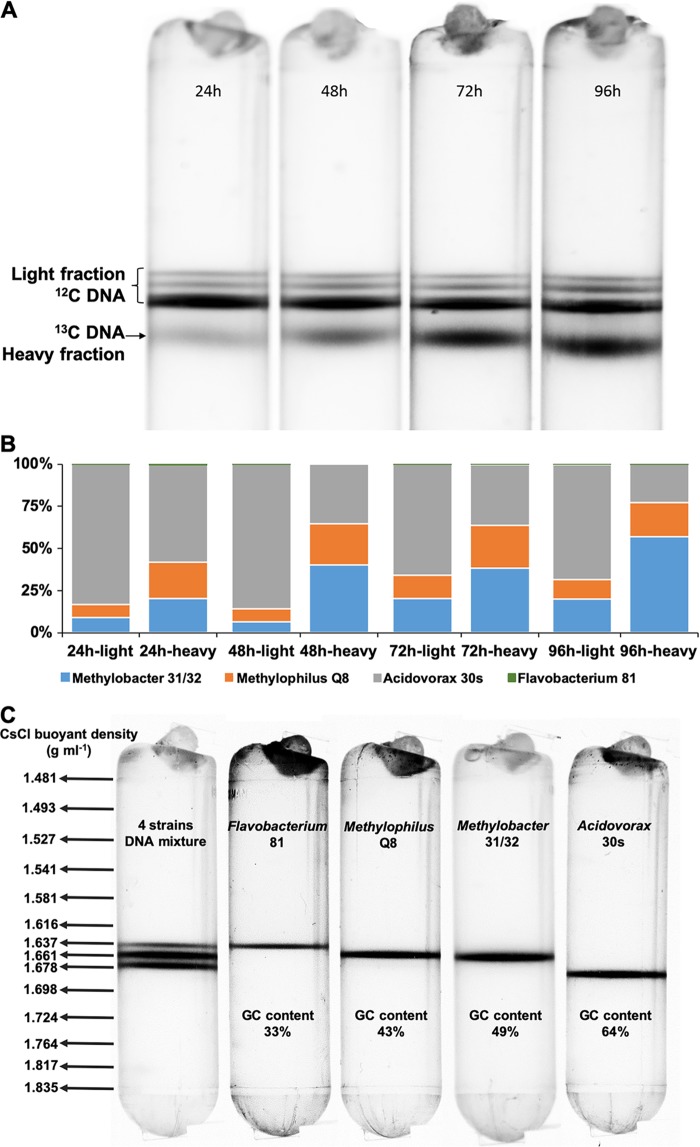
DNA-based stable isotope probing of a synthetic community. (A) Gradual accumulation of ^13^C-labeled DNA from methane over time. (B) 16S rRNA gene fragment profiles of the heavy and light DNA fractions. (C) Separation of low-, medium-, and high-GC DNA in the CsCl gradient, and distribution of buoyant density across the gradient.

To follow the fate of carbon from methane, DNA stable isotope probing (DNA-SIP) was employed. For this experiment, a four-strain coculture was established from *Methylobacter* sp. strain 31/32, *M. methylotrophus* Q8, *Acidovorax* sp. 30s, and *Flavobacterium* sp. 81, and this coculture was incubated with [^13^CH_4_]methane for up to 96 h. Samples for DNA extraction and visualization were taken every 24 h. A gradual accumulation of heavy (^13^C-labeled) DNA was observed over time (Fig. 3A). Sequencing of 16S rRNA gene fragments amplified from each fraction demonstrated that DNA from all four microbes was detectable in the heavy fractions, even though the *Acidovorax* sp. 30s DNA appeared to be overrepresented and *Flavobacterium* sp. 81 DNA appeared to be downrepresented, likely due to amplification biases (Fig. 3B). Note that [^12^C]DNA was represented by three bands (Fig. 3A and C), reflective of the differences in GC values of the respective genomic DNA molecules (Fig. 3C). These DNA-SIP data support carbon transfer from the methanotroph to the partner species in these minimalist synthetic communities.

### Minimalist synthetic communities present model systems for understanding ecosystem processes.

**FIG 4:**
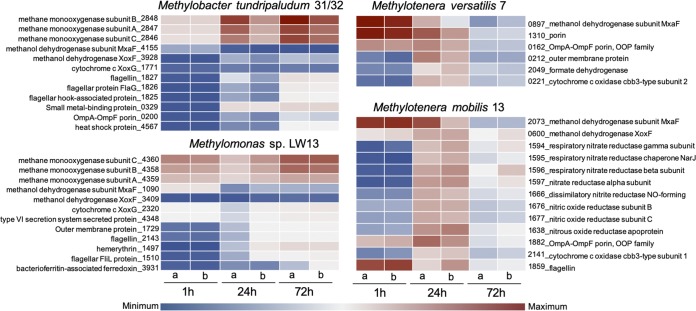
Differential gene expression in major species in response to hypoxia. Heatmaps depict expression of select genes in four major species in a 14-strain synthetic community. Methylobacter tundripaludum 31/32 and *Methylomonas* LW13 are the methanotrophs. *Methylotenera versatilis* 7 and Methylotenera mobilis 13 are nonmethanotrophic methylotrophs. Note that actual expression ranges (minimum to maximum) are different for the *Methylococcaceae* and the *Methylophilaceae*. Complete transcriptomics data are presented in Tables S2 and S3.

As a proof of principle, we employed minimalist synthetic communities of different complexities to investigate specific metabolic process, such as the response to hypoxia and the role of denitrification in communal function. Intriguingly, while in the laboratory, pure cultures of methanotrophs as well as of methane-oxidizing communities perform best when dioxygen is abundant (18, 19, 21), the propensity of these communities to occupy hypoxic niches has been noted, based on culture-independent experiments (8, 12, 16). While the mechanisms for survival and activity under hypoxia remain poorly understood, intriguingly, some *Methylococcaceae* and some *Methylophilaceae* encode functions for either partial or complete respiratory denitrification (22–24), and the role of this metabolic capability in either methane oxidation or in adaptation to hypoxia remains unknown. We here established four-species and 14-species communities to investigate strain-resolved transcriptomic response to hypoxia. Three transcriptomics data sets in two technical replicates were obtained for each community, and the transcriptomes of the oxygenated samples were compared to the transcriptomes of hypoxic samples (Fig. 4 and Tables S2 and S3). When mapped to the respective genomes, not surprisingly, the majority of the transcripts were matched to the methanotroph genomes, as would be expected from cell counts (Fig. 2B), with a significant portion of the transcripts matching the methane monooxygenase genes, as previously reported (13, 25). The *Methylophilaceae* were the second most represented functional guild, with some of the most expressed genes being the *mxaF* genes, encoding the calcium methanol dehydrogenase (MDH) subunit (Fig. 4), while *Flavobacteriales* and *Burkholderiales* showed much lower expression (Tables S2 and S3). Among the highly active species, interesting patterns could be noted; for example, while core genes/pathways revealed similar expression patterns in the microbes of the same functional guild, suggesting that they must be competing for the same substrate (methane or methanol), some of the auxiliary/unique genes were differentially expressed. For example, *Methylomonas* sp. LW13 highly expressed a gene for a type VI secretion system, unique to this organism, and its expression appeared to increase under hypoxia (Fig. 4). It also highly expressed a unique outer membrane protein (1729), while *M. tundripaludum* 31/32 expressed genes encoding different types of porins. While *M. tundripaludum* 31/32 (but not *Methylomonas* sp. LW13) encodes functions for the (partial) respiratory denitrification pathway (24), the expression of the respective genes did not change in response to hypoxia (Tables S2 and S3). Remarkably, *M. mobilis* 13 expressed the entire respiratory denitrification pathway, with maximum gene expression at 24 h after the onset of hypoxia (Fig. 4). All species decreased the expression of the calcium MDH (MxaF), while the expression of the alternative MDH (XoxF) increased in some species and remained unaffected in others. Interestingly, flagellum functions were upregulated in response to hypoxia in *Methylococcaceae*, while they were downregulated in *Methylophilaceae*. Overall, our data demonstrate that differential gene expression in the competing species belonging to the same functional guild can be assessed at high resolution.

10.1128/mBio.01072-19.2 TABLE S2 Normalized transcript counts for the four-species synthetic community. Download Table S2, XLSX file, 1.3 MB. Copyright © 2019 Yu et al. 2019 Yu et al. This content is distributed under the terms of the Creative Commons Attribution 4.0 International license.

10.1128/mBio.01072-19.3 TABLE S3 Normalized transcript counts for the 14-species synthetic community. Download Table S3, XLSX file, 2.4 MB. Copyright © 2019 Yu et al. 2019 Yu et al. This content is distributed under the terms of the Creative Commons Attribution 4.0 International license.

**FIG 5:**
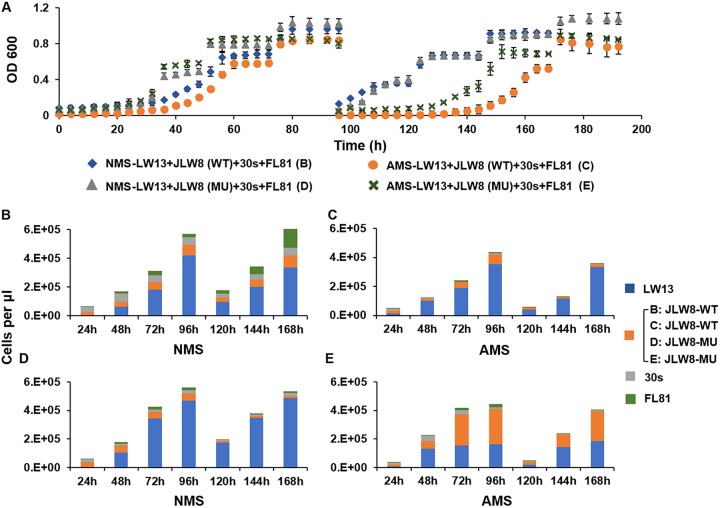
Differential community behavior caused by altered nitrogen metabolism in a community member. (A) Optical densities at a wavelength of 600 nm measured over time. At the 96-h point, cultures were diluted 10-fold in a fresh medium. Error bars indicate standard deviation (*n *=* *3). (B–E) Flow cytometric cell counts taken at 7 time points. WT, wild type (Methylotenera mobilis JLW8); MU, nitrate reductase mutant of *M. mobilis* JLW8 (see reference 26).

We further employed our minimalist synthetic communities to demonstrate how they can assist in dissecting the roles of specific genes/pathways in communal behavior. In this experiment, we questioned whether community living could overcome a deficiency in nitrate metabolism in one of the community members, Methylotenera mobilis JLW8. We previously constructed a mutant of this organism deficient in periplasmic nitrate reductase (Nap), resulting in the loss of its ability to both grow with nitrate and to denitrify (26). We employed this mutant as well as wild-type *M. mobilis* JLW8 in four-species communities cultivated on either nitrate or ammonia as nitrogen sources (Fig. 5). Remarkably, the mutant appeared to be able to maintain its population in nitrate medium over time, even if somewhat less successfully than the wild type (Fig. 5A, B, and D), suggesting that *M. mobilis* could obtain ammonia from other partners to sustain its growth. Interestingly, the mutation in Nap appeared to increase the propensity of *M. mobilis* to form communities compared to the wild type (Fig. 5A, C, and E), suggesting that this loss of function changed the communal behavior of the *Methylophilaceae* partner. While the exact mechanism of such species interaction is yet to be determined, this observation adds new details to the puzzle of Methylotenera species showing preference for nitrate as a nitrogen source (22). This technique can be used for screening other genes for their role in community function.

### Summary.

Understanding the role of microbial community composition in shaping biodiversity-ecosystem function relationships is a challenging goal (27). Synthetic communities have emerged as a prominent model system for dissecting relationships between ecosystem processes and community interactions (28–31). Our focus has been on understanding the communal function in methane oxidation. We previously demonstrated, through isotopic labeling and natural community manipulation in combination with omics analyses, that in our model system Lake Washington sediment, as well as in other environments active in cycling methane, the most prominent active species are representatives of the *Methylococcaceae* (15, 18–20, 32, 33). In addition to *Methylococcaceae*, members of *Methylophilaceae* have been implicated in a role in methane consumption (15, 18–20). We have previously established two-species synthetic communities and demonstrated the differential expression of some key functions in response to communal living (13). In this study, we further increased the complexity of the rationally designed synthetic communities by introducing minor community members, *Burkholderiales* and *Flavobacteriales*, and demonstrated that such communities can perform robustly, suggesting carbon transfer from methanotrophs to nonmethanotrophs beyond methylotrophs. By substituting alternative members of the four specific functional guilds, we demonstrated the flexibility of our platform. As alternative model organisms demonstrate some unique features, for example, the capability of denitrification and alternative stress responses, the respective functions can be dissected by employing organisms with preselected genotypes/phenotypes. We also demonstrated the utility of our approach for connecting organism’s genotype to community living phenotype by substituting a mutant lacking in denitrification for the wild-type phenotype. Overall, we present a proof of a concept in a series of synthetic community manipulations that demonstrates a potential for high-resolution inquiry into the mechanistic details of community function and presents a model for further elucidating the complex relationships and partnerships in communities functionally involved in specific biogeochemical processes. The potential applications of such simplified, genetically defined, and easily controlled model systems range from predictive analyses of greenhouse gas emission mitigation or bioremediation, or even enquiry into microbiome properties relevant to human health.

## MATERIALS AND METHODS

### Isolation of model organisms representing low-abundance persistent species in methane-oxidizing communities.

To test our concept of additional functional guilds as prominent members of methane-oxidizing communities, we carried out isolation experiments, aiming at obtaining pure cultures of *Burkholderiales*, especially Acidovorax species, and *Flavobacteriales*, especially Flavobacterium species, based on our examination of metagenomic data (18, 19). We utilized frozen cultures from prior experiments (18, 19), and we established new cultures, inoculated with frozen sediment, as described before, under both high- and low-dioxygen conditions (18, 19). Dilutions of the resulting cultures were plated onto R2A agar plates (Thermo Fisher Scientific, Waltham, MA, USA) or diluted R2A plates (1/2 and 1/5 dilutions), and plates were incubated at room temperature (approximately 24°C) for up to 2 weeks, until clearly visible colonies appeared. The colonies were examined for shape and color, and representatives were purified through serial transfers onto the same medium. DNA was then isolated, and 16S rRNA genes were sequenced as previously described (22). Strains that appeared most distinct in their phenotypes/genotypes were chosen for further genomic/phenotypic analysis. Genome sequencing was carried out by the DOE Joint Genome Institute (JGI) facility, and genomic data are available through the IMG website (https://img.jgi.doe.gov/). Information on the strains employed in this study, along with the information on previously described Lake Washington sediment isolates, can be found in Table S1. Genome analysis indicated that neither *Burkholderiales* nor *Flavobacteriales* encoded any known methylotrophy functions, and tests for growth on methane or methanol, respectively, were negative (data not shown).

### Synthetic community manipulation.

To investigate the dynamics of pure cultures and cocultures, species listed in Table S1 were mixed in different combinations based on equal optical density at 600 nm (OD_600_) values. Prior to mixing, pure cultures were grown in either nitrate minimal salts (NMS) (34) plus methanol or methylamine (methylotrophs) or in R2A (nonmethylotrophic heterotrophs) liquid medium to late-exponential phase. Cells were collected and resuspended in NMS medium. The created microcosms, along with pure culture controls, were placed into 30-ml tubes sealed with rubber stoppers (Wheaton, Millville, NJ, USA). Each microcosm was incubated under either NMS or ammonia minimal salts (AMS) (34) medium under a 25% methane/75% air (vol/vol) headspace atmosphere. To create this atmosphere, the headspace was flushed with air for 2 min, 5 ml of air was removed using a syringe, and 5 ml of CH_4_ was injected into the headspace. Tubes were incubated with shaking (200 rpm) at 18°C. The headspace gas composition was replenished every 24 h. The OD_600_ measurements were carried out using a Jenway 7300 spectrophotometer (Bibby Scientific, Burlington, NJ, USA) every 4 to 6 h. The values presented represent the results of 3 measurements, reported with a standard error. For experiments involving Methylotenera mobilis JLW8, lanthanum (10 μM) was added to the medium, as this organism relies on lanthanides for growth on methanol (22).

To obtain real-time relative population abundances, cell numbers were determined by flow cytometry. The differences in cell shape and size allowed for separation of all four populations (17). Nine hundred-microliter samples were taken and immediately fixed with 100 μl of a mixture of glutaraldehyde and paraformaldehyde (1.6 and 0.1% final concentrations, respectively) and stored at 4°C. For the analysis, 10 μl of a fixed sample was mixed with 10 μl of SYBR green dye (Thermo Fisher Scientific, Waltham, MA, USA) diluted 1:100 in dimethyl sulfoxide (DMSO) and 0.22-μm filtered NMS medium to a final volume of 830 μl. These samples were incubated for 30 min in the dark at room temperature. Cells were counted with a CyFlow space flow cytometer (Partec, Münster, Germany), with the following parameters: triggering on green fluorescence; all measured parameters, i.e., side scatter (SSC), forward scatter (FSC), green fluorescence analyzed and displayed in log3 or 4; flow rate between 4 and 6 μl/s; and particle analysis rate below 1,000 particles/s.

### DNA-SIP and sequence analysis.

Four model organisms, Methylobacter tundripaludum 31/32, Methylophilus methylotrophus Q8, Acidovorax sp. 30s, and *Flavobacterium* sp. 81, were mixed at equal optical densities. Fifty-milliliter cocultures were grown in NMS medium in 250-ml vials, with 25% ^13^C-labeled methane/75% air (vol/vol) in the headspace for 24, 48, 72, and 96 h, using 3 vials per experiment. Their contents were mixed before DNA extraction. DNA was extracted using the MPBio FastDNA Spin kit for soil (MP Biomedicals, Santa Ana, CA, USA), following the manufacturer’s instructions. The heavy (^13^C-enriched) fractions of DNA were separated from the light (^12^C) fractions by CsCl-SYBR green dye density gradient ultracentrifugation, visualized under UV, and collected and purified following standard procedures, as previously described (15, 20). DNA was sequenced and data were analyzed at MR DNA (Shallowater, TX, USA), using the company’s standard pipelines, essentially as previously described (35).

### Time-resolved metatranscriptomic analysis.

Cocultures (50 ml) were assembled by mixing four or 14 strains at equal optical densities. These were grown in NMS medium with 25% methane/75% air (vol/vol) in the headspace, in duplicate. When cultures reached an OD_600_ of approximately 0.5 (approximately 24 h), samples were flushed with dinitrogen and supplemented with methane (25%), but not air, to create hypoxic conditions. Samples for RNA extraction were taken after 1, 25, and 49 h. Total RNA was extracted as previously described (25), and transcript sequencing was carried out at the JGI, using standard JGI pipelines. Read trimming and adapter removal were carried out using Trimmomatic (version 0.36 [36]). The genomes of the organisms of the four- and the 14-species cocultures were combined to produce respective reference data sets (note that for the 14-species coculture, one genome was not available, so only 13 genomes were used). The trimmed reads were mapped to the reference data set with Bowtie 2 (version 2.3.4 [37]). The abundances of transcripts matching each gene were calculated with HTSeq (version 0.9.1 [38]) and then normalized according to the transcripts per million (TPM) standard, that was, dividing the read counts by the length of each gene in kilobases per million (scaling factor).

### Data availability.

The transcript sequences have been archived with the IMG Genome Portal IMG genome identifiers (IDs) 1125205, 1125208, 1125211, 1125214, 1125217, 1125220, 1125223, 1125226, 1125229, 1125232, 1125235, and 1125238.
