# Supplementary material for: Synthetic Methane-Consuming Communities from a Natural Lake Sediment
Source: mBio. 2019 Jul 23;10(4):e01072-19. doi: 10.1128/mBio.01072-19 (PMC6650549; doi:10.1128/mBio.01072-19)
Supplement: TABLE S1 [file mBio.01072-19-st001.docx]

**Table S1. Strains used in this study, with respective characteristics**

| Organism | Functional category | Growth substrate | Reference |
| --- | --- | --- | --- |
|  | **Methanotrophs** |  |  |
| *Methylomonas* sp. LW13 |  | Methane | 24 |
| *Methylobacter tundripaludum* 31/32 |  | Methane | 24 |
|  | **Non-methanotrophic methylotrophs** |  |  |
| *Methylophilus methylotrophus* Q8 |  | Methanol | 23 |
| *Methylophilus methylotrophus* 42 |  | Methanol | 22 |
| *Methylotenera mobilis* 13 |  | Methanol | 23 |
| *Methylotenera mobilis* JLW8 |  | Methylamine | S1 |
| *Methylotenera mobilis* JLW8 ΔNap |  | Methylamine | 26 |
| *Methylotenera versatilis* 7 |  | Methanol | 23 |
| *Methylotenera* sp. 1P/1 |  | Methanol | 22 |
|  | **Non-methylotrophic heterotrophs** |  |  |
| *Acidovorax* sp. 30s |  | R2A | This study |
| *Acidovorax* sp. 69 |  | R2A | This study |
| *Acidovorax* sp. 107 |  | R2A | This study |
| *Comamonas* sp. 26 |  | R2A | This study |
| *Flavobacterium* sp. Fl |  | R2A | S2 |
| *Flavobacterium* sp. 11 |  | R2A | This study |
| *Flavobacterium* sp. 81 |  | R2A | This study |
| *Flavobacterium* sp. 109 |  | R2A | This study |

**Supplementary References**

S1. Lapidus A, Clum A, Labutti K, Kaluzhnaya MG, Lim S, Beck DA, Glavina Del Rio T, Nolan M, Mavromatis K, Huntemann M, Lucas S, Lidstrom ME, Ivanova N, Chistoserdova L. 2011. Genomes of three methylotrophs from a single niche reveal the genetic and metabolic divergence of the *Methylophilaceae*. *J Bacteriol* **193**:3757-64.

S2. McTaggart TL, Shapiro N, Woyke T, Chistoserdova L. 2015. Draft genomes of two strains of *Flavobacterium* isolated from Lake Washington sediment. *Genome Announc* **3** pii: e01597-14.
